# General commentary: sexual dimorphism for juvenile body weight in lines of chickens selected for 8-week body weight

**DOI:** 10.3389/fphys.2025.1607477

**Published:** 2025-06-11

**Authors:** Colin G. Scanes

**Affiliations:** Department of Biological Sciences, University of Wisconsin Milwaukee, Milwaukee, WI, United States

**Keywords:** sexual dimorphism, chicken, selection, growth, siegel 1

## 1 Introduction

This contribution is to recognize the scientific contribution of Paul Siegel and explore the report from Siegel and Honaker (2025) on sexual dimorphism of chicken. The present discussion explores the putative physiological mechanisms for the consistency of sexual dimorphism in growth rate across 67 generations. Paul Siegel has been conducting research on chicken growth and its genetic control for over 60 years. He is one of the people who established that the growth rate of chickens is highly heritable with the heritability of growth calculated as 0.39 from a study by Siegel (1962) and 0.41 from 176 reports as 0.41 (Siegel, 1962). Recently, he published a paper on the effects of selection for growth over 67 generations (one generation per year) focusing on the effects on sexually dimorphism in growth (Siegel and Honaker, 2025). Birds were selected for either high growth or slow growth (specifically body weight at 8 weeks old). Breeding employed 4 dams for each sire with matings of full- and half-sibs being avoided. Sexually dimorphism of growth was stable over 67 generations selected for growth. This indicates that there is a strong selection constraint for sexually dimorphism of growth and/or that it is a canalized genetic response. Sexually dimorphism of growth was also markedly greater (2.17-fold in the high growth line and 2.51-fold in the slow growth line) at 8-weeks old compared to 4-weeks old (Table 1).

**TABLE 1:** Comparison of sexual dimorphism in 4- and 8 – week-old chickens selected for 8-week-old body weight for 67 generations (calculated from data in Siegel and Honaker, 2025).

	Sexual dimorphism in growth (males minus females) as % of males males	
	4 weeks-old	8-weeks old
Parental generation	11.1	19.1
Generations F_57_-F_67_		
High growth line	8.4	18.2
Low growth line	7.5	18.8

Experimentally, growth is measured as either weight or height/length at one or several time points or the delta increase in weight or height/length (average daily gain or ADG) or expressed as parameters in an equation for growth such as the Gompertz equation. In livestock and poultry growth is most frequently expressed as weight or weight gain. In contrast, human growth is assessed as height (e.g., Gasser et al., 2009). while studies in reptiles employ length; the latter being the distance between snout–vent length in reptiles (e.g., Cox and John-Alder, 2007). Siegel and Honaker (2025) employed body weight at 8 weeks old as their parameters of growth. 

While growth is a change in weight or height/length, a confounding conceptual issue is that there can be sexual dimorphism in mature weight or height/length at sexual maturity or when epiphyseal plates fuse. It is noted that either adult males or females can be larger even in closely related species. For instance, there is opposite sexual adult size dimorphism in lizards (*Sceloporus virgatus*: male < female; *Sceloporus jarrovii*: male > females) (Cox and John-Alder, 2007).

## 2 Sexual dimorphism and growth

It is reasonable to assume that there has been tremendous selection pressure for animals to have the optimal size/weight and growth profile (delta size per unit time) for a specific environment. The corollary is that there will be optimal size/weight together with growth for the food available and other environmental considerations such as predators, temperature, and water availability.

In humans, sex differences in height are only small until puberty (reviewed: Gasser et al., 2009). Similarly, there is greater sexual dimorphism in body weight at 8- compared to 4–weeks of age (Table 1) (Siegel and Honaker, 2025).

## 3 Genetic basis of sexual dimorphism and growth

Sexual dimorphism of growth may have a simple genetic basis. In eutherian mammals, females have two X chromosomes and, consequently, two sets of genes. While one X chromosome is inactivated, some genes escape inactivation and there can be gene dosing (reviewed; Moeser et al., 2022). In birds having ZZ (males) and ZW (females), there is dosage with the Z chromosome gene, Z chromosome gene Doublesex and Mab-3-Related Transcription factor 1 (DMRT1) (Ioannidis et al., 2021; Li et al., 2025; reviewed: Zhang et al., 2023).

## 4 Physiological bases of sexual dimorphism and growth

### 4.1 Sex steroids

It is frequently assumed that the overall mechanism for sexual dimorphism in growth are sex steroids. Sex steroids promote growth in cattle. Castration reduces growth rate in cattle (e.g., Lee et al., 1990; Marti et al., 2013; Li et al., 2022). Moreover, castration depresses circulating concentrations of growth hormone and thyroid hormones and is followed by shifts in microbial fermentation (Li et al., 2022; Shi et al., 2024). Implanting a mixture of androgens and estrogens (trenbolone acetate and estradiol 17β) in increases growth (average daily gain) in steers while reducing protein turnover and the insulin response to glucose (e.g., Ferguson et al., 2023).

There are markedly differences between the effects of androgens on growth in chickens (negative) and turkeys (positive). Growth is either unaffected or tended to be increased by castration in chickens (Fennell and Scanes, 1992a; Chen et al., 2006; Symeon et al., 2010). It is cautioned that body weight gain reflects the aggregate of growth of multiple tissues some or all of which exhibit sexual dimorphism but of different magnitudes and different directions. For instance, while weights of adipose tissue were increased following castration and decreased by androgen replacement, there was no effect of castration on breast muscle but decreases with androgen at physiological concentrations (Fennell and Scanes, 1992a). Moreover, testosterone depressed ADG with the effect overcome by a peripheral androgen blocker (Fennell et al., 1996). Similarly, in female-larger species of reptiles, testosterone reduces growth but increase growth in male - larger species (Duncan et al., 2020). In turkeys, growth and muscle development are enhanced by exogenous androgens (Fennell and Scanes, 1992b) and castration tends to decrease growth and muscle weight (Pierson et al., 1981; Burke and Edwards, 1994).

### 4.2 Hypothalamo-pituitary (growth hormone)-insulin-like growth factor axis

Another underlying assumption is the sexual dimorphism is related to growth hormone-insulin-like growth factor. There are sexually dimorphic patterns for growth hormone secretion, for instance, in humans (e.g., Jessup et al., 2003), rats (e.g., Chowen et al., 1996) and chickens where castration is followed by feminization of GH secretion (Pampori and Shapiro, (1994). The physiological mechanism for SSD involves IGF-1. For instance, castration increases hepatic IGF-1 expression in male *Sceloporus undulatus* while testosterone having no effect (Cox and John-Alder, 2007).

### 4.3 Hypothalamo-pituitary-adrenocortical (HPA) axis

The HPA axis has been related to sexual dimorphism of growth with SNPs in crhr1 that are associated with rates of growth in yellow catfish (Wang et al., 2024). Moreover, there is sexual dimorphism in the effects of corticosteroid-binding globulin (CBG) on hepatic functioning (Toews et al., 2022).

#### 4.3.1 Immune and gastro-intestinal functioning

The extent to which sexual dimorphism is secondary to other sexual differences such as immune or gastro-intestinal is unclear (reviewed; Moeser et al., 2022). For example, there tends to be a larger immune response to *E. coli* or sheep red blood cells in young chickens receiving estradiol with this being blocked by estrogen receptor antagonist (Leiner et al., 1996).

## 5 Discussion

It would be predicted there would be drift in sexual dimorphism over the 67 generations, this was not the case (Table 1) (Siegel and Honaker, 2025). And would suggest that growth and sexual dimorphism are tightly linked. It is speculated that expression of DMRT1 may be, at least partially, responsible for the sexual dimorphism of growth in chickens. What is not known is whether genetic female chickens (ZW) expressing male levels of DMRT1 will grow at male rates or that males with higher levels of DMRT1 expression grow at superior rates. These might be accomplished by selection for DMRT1 expression early in embryonic development or via transgenic approaches.
